# Shifting livelihood strategies in northern Nigeria - extensified production and livelihood diversification amongst Fulani pastoralists

**DOI:** 10.1186/s13570-017-0091-3

**Published:** 2017-07-04

**Authors:** Ayodele O. Majekodunmi, Charles Dongkum, Tok Langs, Alexandra P. M. Shaw, Susan C. Welburn

**Affiliations:** 1 Division of Infection and Pathway Medicine, School of Biomedical Sciences, University of Edinburgh, The Chancellor’s Building, 49 Little France Crescent, Edinburgh, EH16 4SB UK; 2 Trypanosomiasis Department, Nigerian Institute for Trypanosomiasis Research, P.M.B. 03, Vom, Plateau State Nigeria; 3 Veterinary Clinic, National Veterinary Research Institute, P.M.B. 01, Vom, Plateau State Nigeria; 4 Livestock and Poultry Research Centre, College of Basic and Applied Sciences, University of Ghana, P.O. Box LG 25, Legon, Accra, Ghana

**Keywords:** Agro-pastoralism, Livelihood diversification, Vulnerability, Pastoral production, Economic inequality, Sustainability

## Abstract

This paper presents an in-depth investigation of the livelihood strategies of Fulani pastoralists in north central Nigeria. Results show a diversified crop-livestock system aimed at spreading risk and reducing cattle offtake, adapted to natural resource competition and insecurity by extensification, with further diversification into off-farm activities to spread risk, increase livelihood security and capture opportunities. However, significant costs were associated with extensification, and integration of crop and livestock enterprises was limited. Mean total income per capita in the study area was $554 or $1.52/person/day with 42% of households earning less than 1.25/person/day. Income levels were positively correlated with income diversity and price received per animal sold, rather than herd size.

The outcomes of this livelihood strategy were favourable across the whole community, but when individual households are considered, there was evidence of moderate economic inequality in total income, cash income and herd size (Gini coefficient 0.32, 0.35 and 0.43 respectively). The poorest households were quite vulnerable, with low assets, income and income diversity. Implications for sustainability are discussed given the likelihood that the negative trends of reduced access to natural resources and insecurity will continue.

## Introduction

Pastoralism is a livestock-based production system practised in diverse ecosystems across the globe. Pastoral systems across Africa are facing climatic, demographic, economic and socio-political pressures with profound effects on their livelihoods (Sandford 2006, Markakis 2004, Moritz 2008, Blench 2001, FAO 2001b). The changing contexts in which pastoralists operate raise the issue of sustainability of pastoral systems in Africa, particularly in the conflict-prone humid and sub-humid zones populated primarily by cultivators with a very different way of life and limited historical contact with pastoralists. In terms of sustainability of pastoral systems on the African continent, the key issues are mobility, livestock diversity, livelihood diversification and preservation of pastoral traditions and indigenous knowledge (Ayantunde et al. 2000). The extent to which these issues constrain pastoral livelihoods will determine the sustainability of different pastoral systems across the continent.

This paper investigates the livelihoods of Fulani pastoralists in the subhumid zone of north central Nigeria. The specific objectives of the study are as follows: (i) to describe current livelihood strategies and outcomes with emphasis on dynamics and diversity, (ii) to present the current context of risks and vulnerability and (iii) to assess the sustainability of the livelihood strategies employed. The intention is to analyse the dynamics and diversity of these livelihoods in line with the ‘innovations’ discourse, focusing on livelihood adaptations in response to the challenges they face (Azarya et al. 1999, Markakis 2004, Moritz et al. 2009) rather than the ‘crisis’ discourse that has dominated pastoralist research and development in Africa (Hiernaux 1996, Sandford 2006, Thebaud and Batterbury 2001, Turner 2000).

First, the paper describes the crop and livestock enterprises of the study population. Then, wealth groups and livelihood diversity levels are presented. An overview of the wider socio-economic context they operate within follows, as well as specific adaptations and coping strategies. Finally, possible consequences of these changes in livelihood are discussed with special reference to sustainability.

## Study area

This longitudinal study was conducted between April 2012 and March 2013 in six villages (Bokkos, Daffo, Maiyanga, Mangar, Hurti and Tambes) on the Jos plateau, Nigeria. These villages were also part of a longitudinal study on endemic disease control in cattle (Lorusso et al. 2016). There are large numbers of cattle in this area, mostly managed by settled Fulani pastoralists who practise seasonal transhumance in both dry and wet seasons. Six household herds were selected in each of the six villages, so that a total of 36 households were studied. Results from 4 of the 36 sampled households were unreliable and therefore discarded, leaving a final sample size of 32 households for analysis.

Village and household selection were purposive due to the volatile security situation. Since riots in January 2010, there has been fairly persistent insecurity and violence between members of different tribes and religions on the Jos plateau. Bokkos Local Government Area (LGA) was chosen as the study area for this project as it was relatively peaceful and secure. Despite the absence of ethnic/religious violence, armed robberies and cattle thefts affecting both indigenes and Fulani were common in Bokkos LGA, more so than in other areas of the plateau. The high incidence of these crimes was linked to the arrival of displaced Fulani from the Barkin Ladi and Riyom LGAs (centres of protracted violence) and big weekly markets which generate large volumes of cash.

## Methods

A comprehensive livelihood survey was carried out, comprising structured questionnaire interviews with household heads at the beginning of the survey and monthly visits for updates and participatory observations over the next 18 months. Qualitative and quantitative data on income-generating activities, herding and farming practices and off-farm activities were collated to obtain a comprehensive account of the current state of cattle productivity and pastoral livelihoods in the study area. The study also considered whether households had single or multiple income earners and whether these income earners had single or multiple income streams. Male household heads were the major income earners in all cases, controlling the major household assets (land, livestock, etc.) and the income generated from them. Detailed quantitative data on household head income was obtained, but only qualitative data on the income-generating activities of other household members was obtainable. Results from 4 of the 36 sampled households were unreliable and therefore discarded, leaving a final sample size of 32 households.

Cash income from all sources was recorded, as well as household consumption of meat, milk and crops from their own farms to give total income. Cattle and small ruminants owned by households were converted to Tropical Livestock Units (TLUs) at the standard conversion rates of 0.7 for cattle and 0.1 for small ruminants (Otte and Chilonda 2002).

Following the method of McPeak et al. (2012), households were assigned to wealth groups based on the two key determinants of pastoral wealth - livestock and cash income. Thus, households fell into four categories depending on whether they were above or below the sample median for cash income per capita and TLU per capita.

### Data analysis

Pearson’s and Spearman’s correlation tests were applied to income, wealth group and livelihood diversity data to determine significant correlations. Lorenz curves and Gini coefficients were calculated for total income, cash income and TLU to examine wealth distribution and inequality across the study population.

## Results and discussion

### Land tenure and ownership

**Figure 1:**
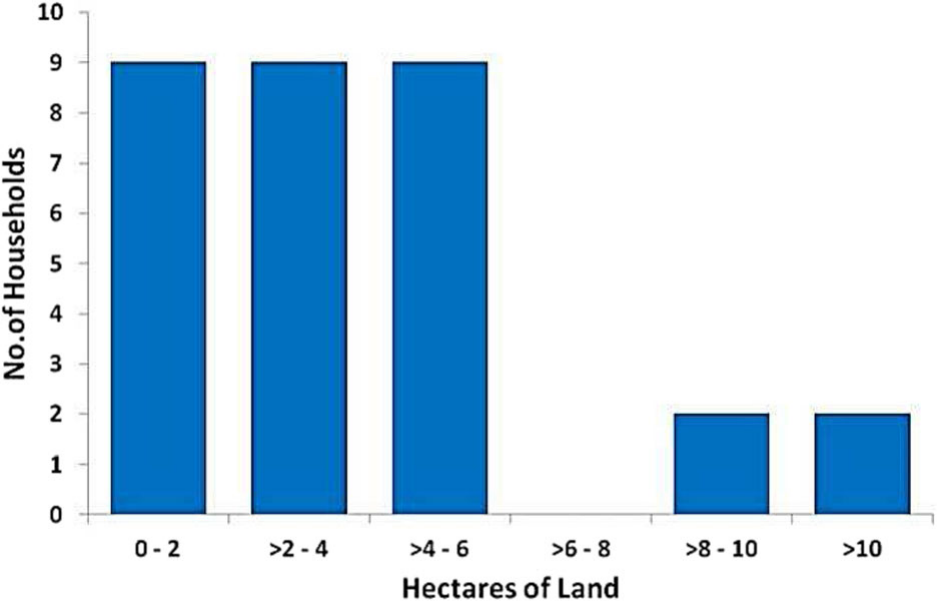
Land holding per household

All but 1 of the 32 households interviewed owned or leased a piece of land, with over 80% having 2- 6 hectares (ha) (Figure 1). Typically, the homestead and cattle enclosures take up ~25% of the land and the rest is farmed. Cattle are grazed exclusively on common land. The times and modes of acquisition of land are laid out in Table 1.

**Table 1:** Land tenure

Mode of acquisition	No. households	% households	Duration of settlement
Inheritance	14	43	35 to 100 years
Purchase	10	30	5 to 63 years
Lease	5	17	7 to 47 years
Occupation	2	7	3 years
Gift	1	3	20 years

Only 30% of households own their land by outright purchase, while 17% lease their plots from the indigenous land-owners. The remaining 53% have less secure tenure, based on occupancy and gifts, both of which can be terminated or revoked. Indeed, several Fulani have been asked to vacate their plots in recent times, including land occupied by their homesteads. The indigenous farmers need more land for several reasons: they now have larger families to support, culture dictates that each son be given a plot of land to farm when he comes of age and there is the natural desire to expand their businesses resulting in increasing fragmentation of existing land and increasing demand for more (Odunuga and Badru 2015). The Fulani understand this and do not necessarily blame the indigenes for these evictions. But there is a growing sense of unease and frustration. If they are asked to leave, where do they go?

### Crop enterprise

**Figure 2:**
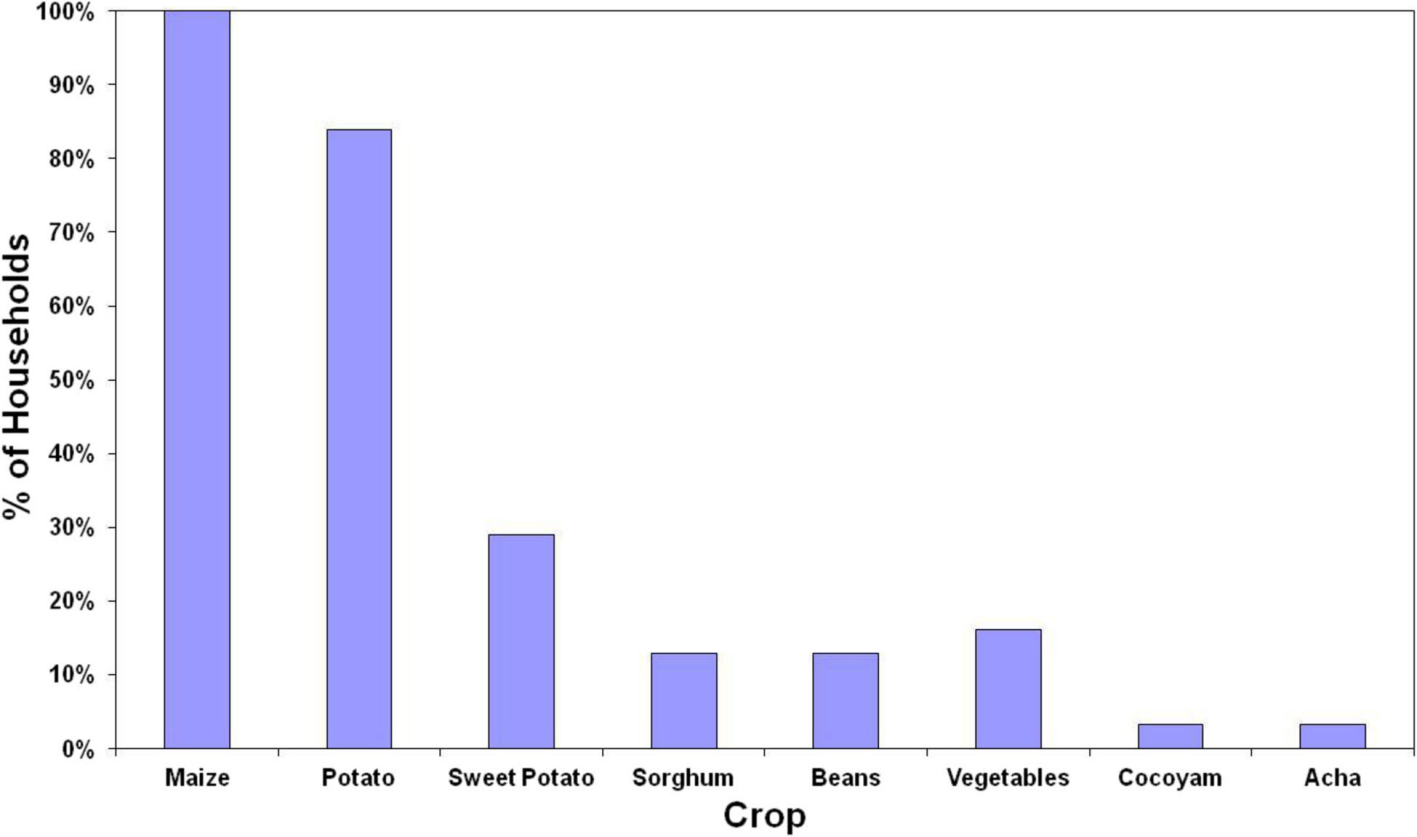
Crops grown by households

Only 50% of households sold crops. Certain staple crops (maize, sorghum and beans) were grown primarily for consumption, while sweet potato, cocoyam and vegetables (carrots, lettuce, cabbages, green peppers) were mostly sold. Potatoes were grown equally for sale and consumption. Annual household income from crops ranged from $100 to $2,000,^1^ with a mean of $867. Figure 2 shows the profile of crops grown.

Amount of land owned was not correlated with family or herd size but was determined by length of settlement and local availability of land. Those who lived in villages with, or settled at a time of, lower population density owned larger plots.

Half of households with 0 to 2 and >2 - 4ha sold crops while the other half consumed their whole harvest. In contrast, 75% of households with more than 4 ha consumed their whole harvest (Table 2). These households grew mostly staples for consumption, representing a decision to feed themselves from their land rather than use it to raise cash. Amongst those with smaller plots, the decision to feed themselves or raise cash from their land was evenly distributed - they cannot grow enough to feed themselves anyway and so are more motivated to get cash income from crops. Those who have large enough plots to feed themselves overwhelmingly choose to do this as it allows them to sell fewer cattle to buy food. Of course, the cultivation of ‘cash crops’ particularly vegetables requires significantly higher investment of inputs (fertiliser, irrigation, pesticides, herbicides) and labour than growing staples. So those with large plots use the opportunity of land as a cost-saving mechanism while those with smaller plots prefer to use it as an investment to raise cash. This cash can then be used to buy more food than the land would have produced in the first place. In both cases, the number of cattle sold to buy food is reduced.

**Table 2:** Household crop income

Land per household (ha)	Households with crop sales (%)	Mean household cash income from crops ($)	Mean household crop consumption^a^ ($)	Mean total income from crops ($)
0 to 2	50	117	2,202	2,319
>2 to 4	56	717	2,351	3,068
>4^b^	25	133	2,911	3,044
All	50	867	2,341	3,208

^a^Calculated using number of hectares per crop, 2012 commodity price per kilogram and yield per hectare (Global-Yield-Gap 2017, FAO 2012)

^b^Fewer than five households in this group sold crops

Labour for cultivation was hired by 94% of households. Of these, 63% left all the work to the farm hands while 31% shared the work equally with them.

It was not possible to collect data on production costs associated with cultivation, but apart from paying for labour and using manure from household cattle, herbicides, insecticides and fertilisers were commonly purchased. Farming labour is certainly problematic for the majority of Fulani households - girls and women do not farm, and so family labour is limited to young men with the requisite skill and strength who can be spared from herding. Most households therefore hire farm hands for the most arduous jobs (clearing, tilling, planting) and may handle weeding and harvesting themselves. Apart from the pull of other activities like herding, there are cultural factors that limit the availability of family labour for cultivation. There is still a certain stigma attached to personal involvement in cultivation - historically, Fulani would have used slave labour to do this, and so there is a high willingness to hire labour rather than do the work themselves. Plateau Fulani mostly practise marriage with seclusion, so women are doubly barred from farming. And the two reasons are linked - when slavery was abolished by the colonial government, well-born women across northern Nigeria refused to do slaves’ work and embraced greater seclusion in protest (Dupire 1970, Hill 2009, Porter 1989).

Amongst those who do sell produce, the mean cash revenue from crops was $867, just 7.9% of mean cash income from livestock (Table 5). So even though all households have diversified into cultivation and half of them sell some produce, crop sales do not contribute significantly to household income. Instead, the food produced for home consumption means that fewer cattle or other livestock have to be sold to buy food, which makes it a worthwhile endeavour. Amount of land owned was positively correlated to cash income from crops (*r* = 0.580, *p* = 0.048) such that households with 2 - 4ha made six times more money from their crop sales than those with 0 to 2 ha. So while the amount of land owned does not determine whether or not households with up to 4 ha sell crops, it does determine how much they make from crop sales.

### Herd size and productivity

Mean herd size (188) was high while the mode and median were 95 and 101 respectively. Herd composition was typical of a breeding herd: equal sex ratios at birth, but high proportion of adult females as males are mostly sold once mature. The management strategy was focused on providing milk and increased cattle numbers for pastoralists and supplying beef to satisfy the high market demand. Productivity of cattle has been analysed in detail in Majekodunmi et al. (2016). To summarise, it was characterised by high births (13.4%), high calving rates (48.8%) and low mortality (5.0%). Thus, high natural herd growth (8.4%) and moderate offtake rates (6.9%) allowed households to maintain herd sizes with a marginal net increase in cattle numbers (1.6%), allowing pastoralists to survive without depleting their herds, despite their significant production costs. Offtake rates lower than those reported in similar production systems (Ducrotoy 2015; Pullan and Grindle 1980) were partly due to good prices for cattle and partly due to livelihood diversification which has reduced reliance on cattle sales for income.

Seventy percent of households owned sheep with a mean flock size of 37. Sheep productivity was characterised by high births (35.0%), high mortality (21.2%) and high offtake (17.8%) (sheep are typically sold or slaughtered in preference to cattle) and an overall negative herd growth. Disease and related mortality remain significant constraints to productivity.

### Milk production and women’s income

Milk production peaked during the wet season when mean offtake was 14.5L/day. Of this, an average of 5.0L was consumed within the household and the remaining 9.5L sold as milk, yoghurt, cheese or butter, in order of importance. The dairy products were either sold to retail customers in the marketplace or collected by wholesale customers from the household at $0.33/L. On average, per household, 855L were sold during the wet season and women’s mean annual income from milk was $282. Women in 88% of households were able to earn money from milk, whereas in 12% of households, all milk offtaken during the wet season was consumed by the household. Mean milk offtake during the dry season was 1.2L/day, usually all for household consumption. In 24% of households, there was no offtake of milk for household consumption during the dry season.

Milk available for sale by women is determined by four factors: cow nutrition and milk yield, offtake, herd splitting for transhumance and household consumption (Niamir 1982). Milking is done by the herdsmen (either a family member or hired herder), and the milk is then handed over to the women of the household. As such, offtake is at the herder’s discretion, who balances the needs of calves with those of the household, often in favour of the former. Transhumance, practised by all households in this study, makes it necessary to split the herd. The majority of cattle are taken away on transhumance, and a few are left behind to provide milk for the household and to save those with infirmities or young calves the stress of the journey. The absence of the majority of the herd for up to nine months in the year further limits the amount of milk available and increases the competition between household consumption and sale. All of these factors have serious implications for women’s income, so much so that women in 12% of households had no milk to sell.

The amounts of milk available for sale in this study were equal to those recorded by Waters-Bayer (1985) in neighbouring Kaduna state when adjusted for herd size - 0.08 L/lactating cow/day (3.7 L/day with a herd of 46 compared to 14.5 L/day with a herd of 188; similar herd composition). Milking was also done by the herdsman in that study.

Women’s main source of income was the sale of milk and other dairy products, although a few engaged in trade (petty trade of food items, keeping small ruminants). Mean annual income from dairy products was equivalent to just 5% of the household head’s income.

### Production costs associated with livestock

**Figure 3:**
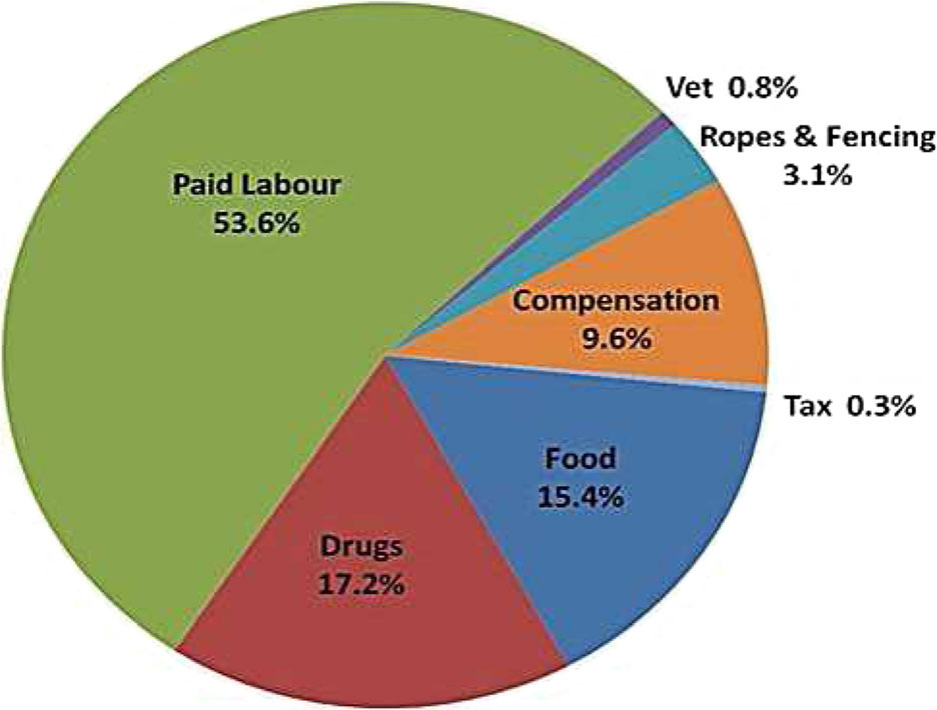
Livestock production costs

Mean annual production costs were $1,892. The cost of non-family labour in cash and kind (animals produced by the herd and given to the herder as a payment) accounts for $1,013 (54% of production costs), followed by drugs $326 (17%), food supplements $291 (15%) and compensation for damaged crops $182 (10%). Only a third of households spent money on fees for animal health workers, which came to $15 per household averaged out over all households (1% of expenditure). The breakdown of costs is illustrated in Figure 3. All the costs were incurred for cattle, with householders not reporting any paid labour or additional costs for small ruminants. Production costs were equivalent to 42% of mean livestock income.

The majority of households (88%) employed extra herdsmen in addition to family labour who were paid at the rate of one heifer (value $308) or $150 cash annually. The average number of hired herdsmen per household was 4. Mode of payment was decided by the employee. Payment in kind (65% of households) was more common than in cash (30%). The exception was one household with a herd of 700 cattle, who hired 15 herdsmen (6 paid in cash and 9 in kind) as several family members were in higher education or paid employment.

Salt lick powder or blocks were the most common food used to supplement free-range grazing, along with small quantities of sorghum/millet bran.

In Table 3, the mean output for the livestock enterprises from all households was estimated. Enterprise output for livestock is calculated by adding the value of home consumption and animals given out to the value of sales, subtracting purchases and adjusting for changes in the value of the herd. This results in an enterprise output of $7,164 for cattle and $252 for sheep, and a total of $7,425. On this basis, the gross margin before deducting forage variable costs would come to $6,546. Livestock keepers only pay for grazing indirectly, when they have to pay fines as compensation. These were included in the total variable costs of $879. The other paid cost, as mentioned above, is permanent labour, valued at $1,013. The only remaining cost is unpaid family labour. No cattle were lent, exchanged or given away.

**Table 3:** Livestock enterprise output using mean household values

	Number	Unit price ($)	Total value ($)
Sales			
Cattle	10	458.00	4,580
Sheep	5	52.31	262
Litres of milk (wet season)^a^	855	0.33	282
Home consumption			
Cattle	2	458.00	916
Sheep	2	52.31	105
Litres of milk (wet season)	450	0.33	149
Litres of milk (dry season)^b^	330	0.33	109
Purchases			0
Cattle	1	−245.90	−246
Change in herd value			0
Cattle	3	458.00	1,374
Sheep	−2	52.31	−105
Total Enterprise Output			7,425

^a^90 days of wet season × 9.5 l sold and 5 l home consumption per day per household

^b^270 days of dry season × 1.2 l home consumption per day per household and no sales

This extra labour is needed because of management changes in response to natural resource conflict, insecurity and uptake of alternative livelihood strategies.

Increased human and animal populations and agricultural expansion have led to competition and conflict over natural resources. Cattle that were entrusted to young boys in the past must now be herded by older, more experienced hands to avoid damage to crops. Avoiding crop damage is more important than ever due to the recent ethno-religious conflicts in north central Nigeria - people are careful not to cause incidents that could spark conflict (Higazi 2011, Majekodunmi et al. 2014). Armed robbery and cattle rustling have also increased across Plateau state, as economies of conflict are established in the wake of violence and insecurity (Higazi 2013, Kwaja 2014, Azeez and Yahaya 2016) - another reason not to entrust grazing cattle to young boys.

The security situation on the Jos plateau and in Nigeria’s Middle Belt has continued to deteriorate since the completion of this work: violent clashes in the long-running conflict in Riyom and Barkin Ladi LGAs spread into Bokkos LGA at the end of March 2013, resulting in the deaths of 18 Ron-Kulere indigenes and 24 Fulani. Over a hundred houses were torched and hundreds of Fulani displaced (Viewpoint 2013, Daily_Trust 2013). Bokkos LGA has now joined the number of conflict-prone areas in Plateau state with uneasy relations between Fulani and indigenes. The Saf Ron-Kulere, paramount traditional ruler of the area, was murdered in 2016 by ‘suspected herdsmen’, further aggravating the situation (Viewpoint 2016). These events are consistent with the general trend of high insecurity in the rural Middle Belt of Nigeria, caused by criminal activities and natural resource conflict. Attacks are becoming more frequent, with 60 recorded throughout 2015 and the same number in the first half of 2016 (Burton 2016).

Fulani have extensified their cattle management as a consequence of natural resource conflict and now spend longer on transhumance: restricted access to natural resources forces them to move their animals in the wet season in addition to the customary dry season transhumance in search of adequate pasture and water (Majekodunmi et al. 2013, Majekodunmi et al. 2014). Uptake of education, paid employment and trade has also increased amongst Fulani youths, reducing the availability of family labour for herding, especially long distance transhumance which is disruptive to other pursuits. These factors have significantly increased demand for experienced herders. The number of hired herders employed by a household was positively correlated with both household size (*r* = 0.663, *p* < 0.001) and herd size (*r* = 0.713, *p* < 0.001). So those with large herds still required more hired herders, even when they had large families.

Hired herders were mostly paid in cattle at the rate of one heifer worth $308 per year of service. This is the preferred mode of payment for the employers as heifers are readily available and they are not forced to sell older cattle to raise cash. Those who wish to be paid in cash only get $150. Unlike many systems across West Africa, milk does not form a part of the hired herders’ remuneration (Agyemang et al. 1997, Shaw et al. 2006, Tonah 2005). Since there are no additional payments in kind, rates of remuneration in cattle seen here are higher than elsewhere in the region (hired herders in Ghana currently get one heifer for every three years of service). It may also be a reflection of the comparative wealth and stability of Nigerian Fulani who have the lowest poverty incidence in West Africa (Majekodunmi et al. 2014, Rass 2006). In this area, both employers and hired herders are Fulani and cattle remain at the owner’s homestead, except when they are taken on transhumance. Thus, rights to milk remain squarely with the women of the household, in contrast to systems where most of the employers are non-Fulani absentee owners with little interest in milk.

Expenditure on drugs for livestock was the second highest cost associated with cattle production. There are many concerns surrounding this practice: incorrect diagnosis, inappropriate drug choice, poor dosage and administration, etc. (Kingsley 2015). Both drugs and healthcare advice are routinely provided by agro-veterinary shopkeepers who often have no veterinary training. Fulani are aware of this, but unlike qualified veterinarians, agro-veterinary shops are available and easily accessible, and they are forced to rely on them. Poor access to qualified veterinary and para-veterinary staff is responsible for the low contribution of treatment fees to production costs (1%).

### Income diversity

Two components of income diversity are considered here: whether households had single or multiple income earners and whether these income earners had single or multiple income streams.

Income diversity of household heads was high with 67% having more than one source of income. Livestock sales were the most important component accounting for 83% of cash income, ahead of crops (7.9%), milk (5.9%) and off-farm activities (3.4%). Household heads pursued a variety of off-farm activities, including driving commercial buses or motorcycles, owning rental property in Jos, teaching Arabic and trading cattle. Table 4 shows that those household heads with two income streams had higher incomes than those with just one or three streams and made most of this income from livestock. This indicates that they were already better off and have diversified to take advantage of available opportunities to improve their livelihoods, reduce risk and vulnerability and improve resilience. In line with this, income diversity was positively correlated with herd size (*r* = 0.497, *p* = 0.013) and land owned (*r* = 0.452, *p* = 0.027). In contrast, the few households with three income streams were forced to diversify to make ends meet - they earned much less from their cattle and crops and a higher percentage of their income from off-farm ventures than the other groups. The high rate of income diversity and increased income is certainly one of the major factors for the achievements of these pastoralists. It contributes to non-depletion of herds and pays for hired labour and other production costs (Adriansen 2006). The proportion of multiple income-earner households was also high at 66%. Only 34% of households were completely dependent on the household head. Of the remaining 66%, 16% had household members engaged in both paid employment and business, 28% in business only and 22% who received remittances from household members in paid employment elsewhere Those in paid employment were mostly teachers and shop workers, and those who were self-employed were mostly traders in cattle, small ruminants and food stuffs, bus drivers and commercial motorcycle operators. Family members who sent remittances were mostly civil servants, drivers and police or army officers.

**Table 4:** Income levels and diversity

Income Sources	Livestock only	Livestock + crops	Livestock + off-farm	All
% households	33	37	17	13
Mean cash income per capita ($)	260	398	444	278
% livestock income	95	78	85	57
% crop income	–	17	–	8
% milk income	5	5	6	4
% off-farm income	–	–	9	32

### Wealth groups

Households were assigned to wealth groups based on the two key determinants of pastoral wealth - livestock and cash income. Thus, households fell into four categories (Table 5) depending on whether they were above or below the sample median for cash income per capita ($330) and TLU per capita (5.1):

**Table 5:** Wealth groups, assets and income (based on McPeak, Little and Doss 2012)

	All	High livestock, high cash	High livestock, low cash	Low livestock, high cash	Low livestock, low cash
% of households	100	17	33	33	17
Land (ha)	3.9	3.4	4.4	3.6	3.9
Household size	18	22	24	12	13
Tropical Livestock Units	141	224	264	47	42
Tropical Livestock Units per capita	6.4	9.3	9.4	4.1	3.1
Cash income per capita ($)	345	434	185	578	121
Total income per capita ($)	554	619	371	863	266
Mean price per animal sold ($)	416	429	380	513	311
Livestock cash (%)	83	86	88	76	80
Milk cash (%)	6	5	6	4	4
Crop cash (%)	8	9	2	10	7
Other cash (%)	3	0	4	10	9

The high livestock, high cash group was characterised by large household size and income exclusively from livestock and crops with no off-farm diversification. The high livestock, low cash group also had large households, with income from livestock, crops and off-farm sources. However, they were slightly more dependent on livestock sales and made less cash per animal sold (Table 5).

The low livestock, high cash group was characterised by smaller household sizes, the highest price per animal sold and the most income diversity, earning higher proportions of income from non-livestock sources than the other groups. This group also contained the majority of households earning cash from livestock and off-farm enterprises. The low livestock, low cash group also had smaller households and a higher income share from non-livestock sources. However, they made the least amount of cash per animal sold and were slightly more dependent on livestock sales.

TLU was correlated with family size (*r* = 0.933, *p* < 0.001), which explains the clear difference in household size between the high livestock and low livestock groups. There are two reasons for this correlation: first, larger households tend to have older household heads who have had more time and opportunities (such as marriages) to accumulate livestock. The second reason is the existence of ‘super households’ - large extended families made up of grown sons/brothers and their own families who would ordinarily have split into independent households herds but who still live as one family and manage their livestock as a single herd. Such households are found in situations where pre-inheritance (common amongst Fulani pastoralists) is delayed by the household head who retains direct control over the herd, or brothers are dependent on each other for the management of their animals, e.g. when one or more of them is in higher education or employment. Examples of both were found within the study households.

Neither amount of land owned nor village/location were correlated with wealth groups or the attributes measured.

Livestock sales remain the primary source of cash income which was positively correlated, not with TLU but with the price received per animal sold (*r* = 0.607, *p* = 0.002), i.e. those who got the best prices for their cattle earned the highest cash income. In fact, the group with the highest cash income was the low livestock, high cash group which got the highest prices per animal sold.

On average, household consumption accounted for just 38% of total income, while cash income accounted for 62%. Consequently, total income was also slightly correlated with price received per animal sold (*r* = 0.404, *p* = 0.05) in addition to its correlation with income diversity (*r* = 0.499, *p* = 0.013). Those who had multiple income streams had a higher total income. Again, the low livestock, high cash group records the highest total income as it shows the most income diversity.

The difference in price received per animal sold was not significantly correlated with village/location and so cannot be attributed to differences in local market opportunities. Instead, it may be linked to the higher level of diversification into off-farm enterprises seen in the low livestock, high cash group. They may be more exposed to markets due to their involvement in off-farm activities, have better knowledge of prices and preferences further down the value chain and more money for inputs to achieve those standards.

Thus, so we see a two-way relationship between income diversity and income levels. Those with more land, livestock and cash are more likely to diversify their income by investing surplus (pull factor), which leads to higher total income. Those with less land, livestock and cash are also more prone to diversification driven by the need to make ends meet (push factor) which leads to lower total income. Those with intermediate levels of these assets had lower income diversity and intermediate level incomes.

The amount of land and livestock owned on their own were not correlated with either cash or total income. Instead, income was determined by the ability to generate cash from these assets and their push or pull effect on income diversity.

### Inequality and poverty

**Figure 4:**
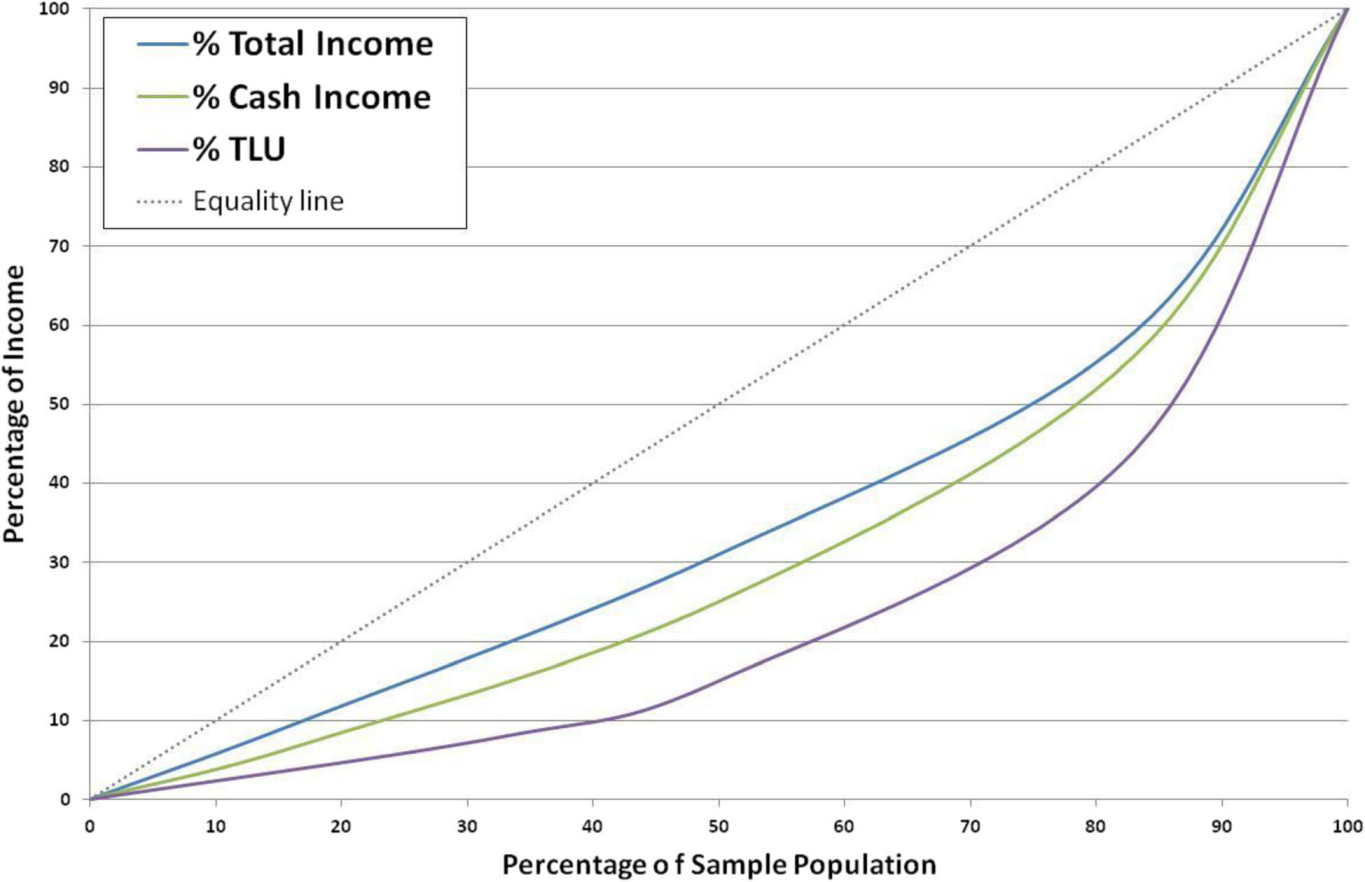
Lorenz curves of the distribution of total income, cash income and TLU

Figure 4 shows that 50% of the population in the higher cash group control 75% of the cash income, while 50% in the higher livestock group control 85% of the TLUs. The Lorenz curve shows inequality in all three measures, with Gini coefficients of 0.32 for total income, 0.35 for cash income and 0.43 for TLU. Thus, income inequality in this sample was lower than the national average of 0.43 (World-Bank 2014) and almost half the recorded levels amongst East African pastoralists (cash income 0.56, total income 0.68, TLU 0.64) (McPeak et al. 2012).

Mean total income per capita and daily income per capita in the study population were $554 and $1.52, respectively, slightly higher than the $495 and $1.40 average for rural households in Nigeria (World-Bank 2014). Forty-two percent of households in this study fall below the poverty threshold of $1.25/person/day, less than the 53% national poverty incidence (World-Bank, 2014).

Overall, results show moderate levels of inequality in terms of land ownership, income, TLU and income diversity. The poorest households in the low livestock, low cash category have a mean daily per capita income of just $0.73, well below the absolute poverty line ($1/person/day) and are therefore very vulnerable.

### Integration and adaptation^2^

Fulani on the Jos Plateau are settled transhumant agro-pastoralists with mixed farms of crops, cattle and sheep. The exchange of resources such as dung, draught and crop residues on mixed farms takes place in differing degrees depending on the availability of land, labour and capital. The central exchange in integrated crop-livestock farming is manure and animal traction for the fields and crop residues for the animals. This recycling of resources increases the efficiency of the mixed farm and reduces labour and capital expenditure requirements. Due to the highly extensive production system (free range grazing, transhumance for nine months in the year), only 30% of manure is collected and only some of this is used on their own fields - the rest is given away to neighbours. Recycling of crop residues for animal feed is quite low, despite their critical role as a dry season feed source - settled herds with access to crop residues are more likely to meet their nutritional requirements and improve their productivity than transhumant cattle (FAO 2001a, van Raay and de Leeuw 1974). The switch from millet and sorghum to maize as the dominant cereal crop in the 1970s has had serious consequences for the availability and use of crop residues as animal feed. First, maize has a low grain to fodder ratio (2) compared to millet (3.8) and sorghum (3.4), so it produces a smaller volume of crop residues (Powell and Bayer 1985, Savadogo et al. 1999). Secondly, in the humid and sub-humid zones, short-duration varieties of maize are preferred and often intercropped with late-maturing crops (mostly potato on the Jos Plateau). Thus, cattle do not have access to standing maize stalks until after the potatoes are harvested, and by that time, they have lost most of their nutritional value (Jabbar 1996, Jabbar et al. 1995, Powell 1986). Collection of crop residues from fields to be fed to animals is rare in this area. Acha (white fonio, *Digitaria exilis*), the original staple crop of the Jos plateau, produces excellent fodder for livestock, but it has also been largely replaced by maize. Former arrangements for free access to crop residues in fields have also declined, partly owing to less cordial relationships between pastoralists and farmers, partly due to private use of crop residues as many farmers now own cattle and small ruminants. Insecure land rights for pastoralists and the impracticality of animal traction on the Jos Plateau (due to the topography) remain barriers to further integration.

According to the classification of Schiere et al. (2002), the mixed farming practised here is a diversified rather than an integrated system, characterised by reuse of dung, manual rather than mechanised labour, outfield grazing, high ratio of outfield to infield resource use, low use of crop residues, exchange of dung and crop residues between farms (with neighbouring crop farmers) as well as within the farm, low milk/meat output per animal, animals viewed as savings rather than commodities and low attention to conservation of the resource base (Schiere et al. 2002). Diversified systems are combinations of specialised subsystems that coexist almost independently. They aim to reduce risk rather than to recycle resources. Such diversified systems are commonly found in areas with relatively abundant natural resources where labour and capital are low and any shortage in land is overcome by migration or transhumance.

Plateau Fulani made the shift from pastoral to agro-pastoral production over 100 years ago, motivated by abundant natural resources in the area, entry into the cash economy and the desire to access social amenities (Awogbade 1979). Further shifts from agro-pastoral systems to integrated crop/livestock systems tend to be instigated by increased human population densities, reduced access to natural resources and increases in services and markets (Herrero et al. 2009, Hobbs et al. 2008). All of these conditions are met within the study area, but these Fulani have responded differently, by extensification and diversification. There have been several different responses to increased population and land pressure by Fulani populations across West Africa as shown in Table 6. These varied responses show dynamic adaptations and combinations of adaptations to suit prevailing conditions.

**Table 6:** Livelihood adaptations of agro-pastoral populations in West Africa

Location	Specialisation	Extensification	Intensification	Market integration	Diversification
Senegal (Adriansen 2006)	Abandoned cultivation	Use boreholes and watering tubes to exploit more rangeland		High capital and labour investment; dramatic changes in herd composition to fit market demand: high proportions of small ruminants for Eid al Adha and all beef rather than milk herds of cattle	High engagement in off-farm enterprise, including large-scale livestock trading
Mali (Ramisch 1999)			Integrated mixed farming		
Cameroon (Moritz 2008)		Long-range transhumance	Stall-feeding industrial cottonseed cake and crop residues	High integration with urban markets which makes the increased labour and cost of stall-feeding worthwhile	
Ivory coast (Diallo 2001, Tonah 2003)					
Niger (Ayantunde et al. 2000, La Rovere et al. 2005)		Long-range transhumance	Night grazing, integrated mixed farming		
Nigeria This study		Long-range transhumance			Significant investment in off-farm enterprises

Although they are agro-pastoralists, Plateau Fulani are still fairly specialised cattle keepers. Their crop and livestock enterprises remain distinct, with extra labour required to keep up with both. They have not increased the level of integration or intensification of their production system in response to dwindling access to natural resources. Instead, they have extensified livestock production and use their capital to subsidise continuation of this system by hiring labour and diversifying into off-farm activities. However, the capacity to hire labour is an important feature of the better-off in society. If hiring labour provides the freedom to pursue diversification, then it should be viewed as positive. This particular model of agro-pastoralism has risen in adaptation to prevailing conditions and seems well suited to this environment.

### Market integration

Both crop and livestock enterprises show some influence of market integration. However, this influence is not strong, and subsistence is still the main goal: livestock production is still mainly for accumulation and risk spreading, and animals are still sold only to raise cash for immediate needs. Small ruminant production has not changed to take advantage of the huge seasonal market opportunities offered by Eid al Adha.

The levels of market integration are far below what is possible considering the huge domestic demand and level of imports of both live cattle and beef in Nigeria (Benard et al. 2010). Other players have stepped in to supply the shortfall - corporate investors, foreign companies, and private business people attracted by the rising demand for animal products and national focus on agribusiness. These competitors run intensive farms and produce and sell beef and live cattle, often of better quality and produced closer to the southern consumer markets. There is also stiff competition lower down the value chain: in urban areas, butchers who buy mostly from pastoralists compete with supermarkets supplied by their competitors, which further reduces their market share (Euromonitor-International 2015, The-Economist 2013).

## Conclusions

The results of this study show a diversified crop-livestock system aimed at spreading risk and reducing cattle offtake, adapted to natural resource competition and insecurity by extensification, with further diversification into off-farm activities to spread risk, increase livelihood security and capture opportunities. This livelihood strategy is well suited to prevailing conditions in the Plateau but is not without cost. The outcomes of this livelihood strategy are quite favourable when considered across the whole community, leading to good productivity in cattle, high incomes compared to the national average and high levels of income diversity amongst household heads as well as multiple earner households. However, there are moderate levels of inequality within the sample, and a proportion of the population is quite vulnerable, with low assets, income and livelihood diversity. Security and access to natural resources are likely to get worse over time, and extensification may not be sustainable. Economic inequality is also likely to continue rising with consequent increases in the vulnerability of poorer pastoralists. It remains to be seen how this group of pastoralists will cope with further pressures to their production system and increasing market demand and competition.

## Footnotes

1 Foreign exchange rate for 2012: 1.0 USD = 165.1 NGN

2 Intensification and extensification as used here refer to increased or decreased capital inputs and productivity per land unit (Moritz et al. 2009)
